# Anagrelide-Induced Supraventricular Tachycardia: A Case Report

**DOI:** 10.7759/cureus.26119

**Published:** 2022-06-20

**Authors:** Faraz Badar, Hayder Azeez, Zeinab Abdulrahman, Aqsa Ashraf, Asma Iftikhar

**Affiliations:** 1 Internal Medicine, Northwell Health, Port Jefferson, USA; 2 Internal Medicine, Zucker School of Medicine/Northwell Health, Port Jefferson, USA; 3 Pulmonary and Critical Care Medicine, Northwell Health, Port Jefferson, USA

**Keywords:** jak-2: janus kinase, myeloproliferative, cardiac arrythmia, thrombocythemia, anagrelide

## Abstract

Anagrelide is an inhibitor of the phosphodiesterase-3 (PDE-3) enzyme that suppresses megakaryocytes; hence it is used in the treatment of essential thrombocythemia. Anagrelide can cause positive inotropic and chronotropic effects on the cardiovascular system. Its cardiovascular side effects are rare and include palpitations, tachyarrhythmias, cardiomyopathy, angina, and heart failure. We report the case of a 71-year-old female who presented with sudden onset chest pain. Her only outpatient medications included anagrelide and aspirin. She was found to have supraventricular tachycardia (SVT) with aberrancy that responded to beta-blockers. The chest X-ray, computed tomography angiogram (CTA), and echocardiogram were unremarkable. Her arrhythmia may be attributed to the anagrelide in the absence of any cardiovascular findings.

## Introduction

Essential thrombocythemia (ET) is a myeloproliferative neoplasm that can present with a spectrum of symptoms, ranging from flushing, headache, and visual disturbances due to vasculogenesis or abnormal bleeding and clotting due to platelet dysfunction [1]. Anagrelide is an oral antiplatelet drug commonly used in the treatment of ET. Its mechanism of action involves inhibition of phosphodiesterase-3 (PDE-3) enzyme, which in turn prevents the maturation of megakaryocytes to platelets. Although a clear correlation between the administration of anagrelide and these events has not been well established, studies demonstrate that the adverse events disappear after anagrelide is discontinued [1]. Known to be potentially cardiotoxic in rare instances due to arrhythmogenesis, we present the case of a 71-year-old female found to develop supraventricular tachycardia (SVT) while using anagrelide.

## Case presentation

A 71-year-old female presented to the emergency room with sudden onset substernal chest pain that started five to six hours previous while driving to work. The pain was episodic in nature, with each episode lasting approximately two minutes, described as 5/10 in intensity, pressure-like in quality, non-radiating, without aggravating or relieving factors, and associated with palpitations, dizziness, lightheadedness as well as arm and neck numbness. A review of systems was negative for dyspnea.

The patient had a history of Janus kinase 2 (JAK2) mutation-negative, calreticulin gene (CALR) mutation-positive essential thrombocythemia, for which she was on aspirin 81mg once daily, anagrelide 0.5mg in the morning and 1mg in the evening. She was a lifetime non-smoker with no history of alcohol or illicit drug use. There was no reported family history of sudden cardiac death.

Initial vital signs were temperature: 98.1 Fahrenheit, heart rate 77 beats/minute, blood pressure 154/56 mmHg, respiratory rate 20 breaths/minute, and oxygen saturation 99% on room air. On general exam, the patient was awake, conversant, and in no acute distress. There was no reproducible chest pain on palpation. Precordial auscultation revealed S1+S2 with irregular rhythm and no murmurs. No jugular vein distension was seen, and the lungs were clear to auscultation bilaterally with an absence of any added sounds. No focal neurological deficits were appreciated, and lower extremities showed no edema.

**Figure 1:**
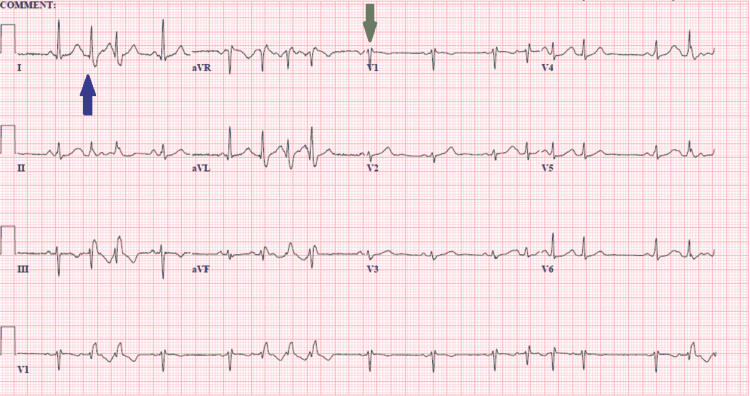
ECG on admission Sinus rhythm with frequent and consecutive premature atrial complexes (marked with blue arrow in lead 1; however can be seen in other leads as well). rSR' pattern in lead V1 (green arrow). QTc: 539ms.

The first ECG showed normal sinus rhythm with atrial premature complexes and right bundle branch block (RBBB). QTc was prolonged at 539ms (Figure 1).

**Figure 2:**
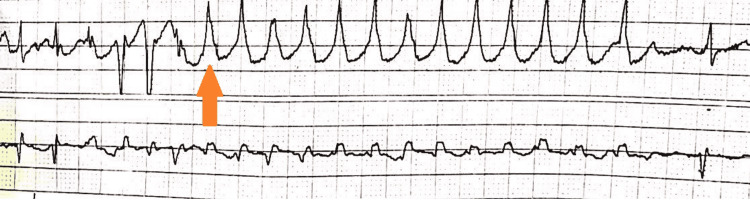
Telemetry strips were concerning for ventricular tachycardia (orange arrow)

Additionally, bedside telemonitoring during the initial encounter was positive for frequent short runs of wide complex tachycardia up to 150 beats/minute (Figure 2).

Complete blood count showed mild anemia (at baseline) and normal platelet count. Serial troponins were unremarkable, and so was N-terminal prohormone of brain natriuretic peptide (pro-BNP). The metabolic panel and thyroid-stimulating hormone (TSH) were completely normal (Table 1),

**Table 1:** Complete blood count showed mild anemia (at baseline) and normal platelet count. Serial troponins were unremarkable and so was pro-BNP. The metabolic panel and TSH were normal. WBC: white blood count, BUN: blood urea nitrogen, pro-BNP: N-terminal prohormone of brain natriuretic peptide, TSH: thyroid-stimulating hormone, CPK: creatine phosphokinase

Hematology		
Name	Result	Reference range
WBC	5.85	3.5 - 10.8 K/ul
Hemoglobin	10.2	11.5 - 15.5 g/dl
Hematocrit	33.3	34.5 - 45.0 %
Platelet count	303	150 - 400 K/ul
General chemistry		
Name	Result	Reference Range
Sodium	141	136- 145 mmol/L
Potassium	4.3	3.3 - 5.1 mmol/L
Chloride	105	98 - 107 mmol/L
Bicarbonate	25	22 - 29 mmol/L
BUN	19	8 - 23 mg/dl
Creatinine	0.79	0.7 - 1.2 mg/dl
Glucose	94	74 - 109 mg/dl
Magnesium	2.1	1.6 - 2.6 mg/dl
Phosphorus	3.1	2.5 - 4.5 mg/dl
pro-BNP	164	1 - 125 pg/ml
TSH	0.729	0.27 - 4.2 uIU/ml
Cardiac enzymes		
Name	Result	Reference Range
Troponin T	13 → 13 → 19	<14 ng/L
CPK	141	120 - 180 U/L

Chest X-ray showed normal cardiac and lung morphology. A pulmonary embolism was ruled out on a computed tomography angiogram (CTA) of the chest.

**Figure 3:**
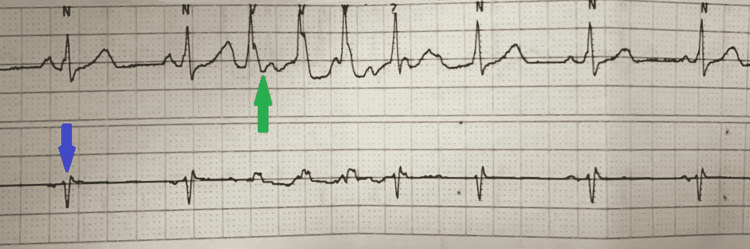
Telemetry strips P waves can be seen preceding the wide complex beats (green arrow). Additionally, the lower lead looks typical for RBBB (blue arrow). RBBB: Right Bundle Branch Block.

Zoll defibrillators were placed immediately, and STAT metoprolol 5mg IV push was given once. Electrophysiology was consulted, and although it was felt that telemonitoring was concerning for arrhythmia originating from the ventricles due to wide QRS morphology, there was clear rSR' consistent with RBBB aberrancy during these runs of wide complex rhythm (Figure 3).

A working diagnosis of supraventricular tachycardia (SVT) with aberrancy was therefore established, and a beta-blockade was initiated to suppress the SVT. The patient received metoprolol tartrate 50mg oral every eight hours.

**Figure 4:**
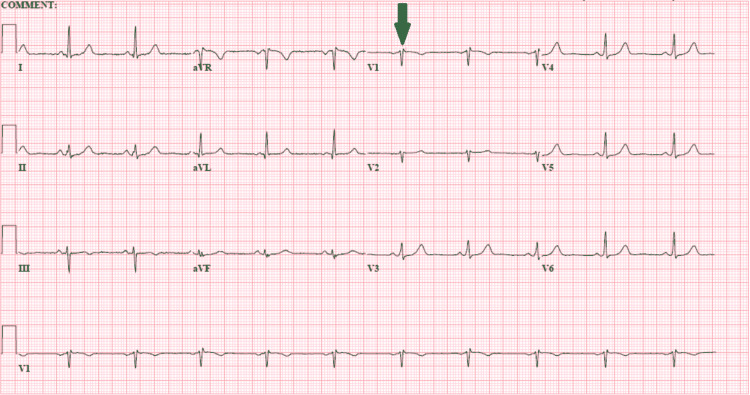
ECG after initiation of beta blockade Normal sinus rhythm with unchanged rSR' pattern in lead V1 (green arrow). Previously seen premature atrial complexes have resolved. QTc shortened to 432ms.

An echocardiogram ruled out structural or wall motion abnormalities. Normal left ventricular systolic function was seen with an ejection fraction of 64%. There were trace valvular abnormalities. The patient's symptoms resolved, and a repeat ECG showed normal sinus rhythm with unchanged rSR' pattern and resolution of previously seen premature atrial complexes (Figure 4).

Cardiology recommended workup for ischemia will be unnecessary. 

Although discontinuation of the drug was strongly considered, it was decided against in light of the prolonged history of usage and prompt resolution of arrhythmic activity. The patient was discharged on sustained-release metoprolol 150mg PO once daily with close outpatient electrophysiology and hematology follow-up.

## Discussion

Anagrelide is an oral phosphodiesterase-3 (PDE-3) enzyme inhibitor used as cytoreductive therapy in myeloproliferative disorders, mainly essential thrombocythemia, due to its ability to prevent the maturation of megakaryocytes and hence effectively reduce platelet counts [2, 3, 4, 5]. Common toxicities of anagrelide are thought to be from vasodilatory effects of PDE-3 inhibition, and these include nausea, diarrhea, headache, and fluid retention. However, PDE3 inhibition also leads to increased intracellular levels of cyclic AMP and intracellular calcium in the cardiac myocytes, which in turn enhances inotropy and chronotropy. These effects can be cardiotoxic by manifesting as arrhythmias [6, 7], cardiomyopathy [8, 9], and heart failure [10, 11]. This side effect profile appears to be dose-related and more pronounced in the elderly population. Reports advise caution if anagrelide is used in patients with prior history of cardiac pathology to prevent decompensation [12]. Prompt assessment, especially via EKG, telemonitoring as well as an echocardiography should be performed for possible arrhythmias or heart failure. Our patient was unique in the sense that anagrelide appeared to be the causative agent of new-onset arrhythmia in the absence of any underlying cardiac disease. For younger patients with ET who have a long life expectancy, the long-term benefits of controlling essential thrombocythemia (ET) with anagrelide should be weighed against its potential cardiovascular risks.

Table 2 summarizes cases of anagrelide-induced cardiotoxicity found in literature.

**Table 2:** Cases of anagrelide-induced cardiotoxicity PCI - percutaneous coronary intervention

Clinical features	Cardiac side effects	References
A 78-year-old woman with a history of hypertension, dyslipidemia, and essential thrombocythaemia presented with syncope.	Non-sustained ventricular arrhythmia	Rodriguez Ziccardi et al. [6] (2018)
A 50-year-old woman diagnosed with essential thrombocythemia (ET) in 1993 has been treated with hydroxyurea. Symptoms like paresthesia of the fingers and acrocyanosis were ameliorated after treatment. She started to take low-dose aspirin and sublingual nitroglycerine occasionally for intermittent chest pain since 2001.	Acute coronary artery disease	Lin et al. [13] (2007)
A 48-year-old woman with polycythemia vera developed cardiotoxicity manifested by congestive heart failure and palpitations.	Cardiomyopathy	James CW [14] (2000)
A 30-year-old woman was admitted to our primary-PCI center for an acute retrosternal chest pain associated with dyspnea.	Inverted Takotsubo cardiomyopathy	Dziewierz et al. [15] (2012)
A 75-year-old woman presented with acute chest pain.	Atypical Takotsubo syndrome	Proietti et al. [9] (2009)
A 30-year-old female patient presented to the emergency department with dyspnea on exertion and severe left anterior chest pain.	Acute myocardial infarction	Lim et al. [16] (2010)
A 34-year-old male has been presented with palpitation, pedal edema, and increased abdominal girth with exertional dyspnea.	High-output heart failure	Engel et al. [17] (2005)
A 48-year-old male presented with exertional angina.	Prinzmetal angina	Luminita et al. [18] (2018 )
A 70-year-old female patient with polycythemia vera admitted to hospital because of severe dyspnea and systemic edema.	Pulmonary hypertension	Sumimoto et al. [19] (2019 )

For younger patients with ET who have a long life expectancy, the long-term benefits of controlling ET with anagrelide should be weighed against its potential cardiovascular risks.

## Conclusions

Cardiovascular complications associated with the use of anagrelide are rare. Our case report highlights an important side effect of this therapy. Clinical suspicion should be high if patients using this medication experience cardiac symptoms. Caution is paramount, especially when usage is indicated in older age or with known cardiac disease. Baseline testing prior to treatment initiation, as well as periodic monitoring during therapy, should be utilized to prevent any life-threatening complications. Discontinuation of therapy needs to be strongly considered in case of positive findings.
